# *oceandatr*: An R Package to Acquire and Process Geospatial Ocean Data

**DOI:** 10.1002/ece3.74211

**Published:** 2026-08-21

**Authors:** Jason Flower, Echelle S. Burns, Daniel C. Dunn, Andy Estep, Jason D. Everett, Jeffrey O. Hanson, Sarah E. Lester, Anthony J. Richardson

**Affiliations:** ^1^ Marine Science Institute University of California, Santa Barbara Santa Barbara California USA; ^2^ Bren School of Environmental Science & Management University of California, Santa Barbara Santa Barbara California USA; ^3^ Environmental Markets Lab University of California, Santa Barbara Santa Barbara California USA; ^4^ School of Environment The University of Queensland Brisbane Queensland Australia; ^5^ Centre for Biodiversity and Conservation Science (CBCS), The University of Queensland Brisbane Queensland Australia; ^6^ Waitt Institute La Jolla California USA; ^7^ Commonwealth Scientific and Industrial Research Organization (CSIRO) Environment, Queensland Biosciences Precinct (QBP) St Lucia Queensland Australia; ^8^ Centre for Marine Science and Innovation (CMSI), The University of New South Wales Sydney New South Wales Australia; ^9^ Department of Biology Carleton University Ottawa Ontario Canada; ^10^ Department of Biological Science Florida State University Tallahassee Florida USA

**Keywords:** conservation planning, geospatial data, gridded data, marine, ocean, R package

## Abstract

Maps and spatial models inform natural resource management and policy decisions. However, the geospatial data needed for these decisions are often scattered across websites and services, and typically require technical skills to download, process and analyze. The R programming language is widely used in environmental science, and while there are some R packages available for obtaining terrestrial geospatial data, those for marine data are limited to specific datasets. To fill this gap, we introduce the *oceandatr* R package, an interface that provides streamlined access to a suite of global ocean data. It provides functions for acquiring and processing many data sources—including bathymetry, geomorphology, ecological, and human use data—and returns these in several grid‐based formats. The gridded output data can be used directly in modeling, spatial analysis and spatial planning. While *oceandatr* accesses marine data, the functions for retrieving boundaries and gridding data can also be used in other contexts. We outline uses of *oceandatr* and present two case studies where we use *oceandatr* to access and process data for a spatial planning application and a spatial model of fishing effort. *Oceandatr* simplifies the process of geospatial analysis for marine researchers and spatial planners, reducing technical barriers and supporting evidence‐based decision making.

## Graphical abstract

We introduce the *oceandatr* R package, an interface that provides functions for acquiring and processing marine data sources—including bathymetry, geomorphology, ecological, and human use data—and returns these in several grid‐based formats. We outline uses of *oceandatr*, such as modeling and spatial analysis, and present two case studies where *oceandatr* is used to acquire, process, and standardize data for a spatial planning and spatial modeling application.

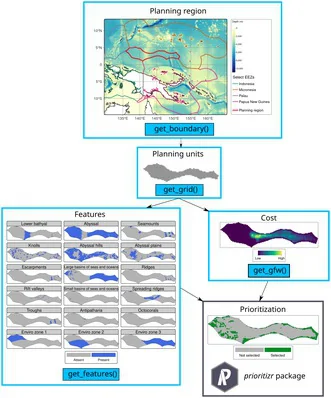

## 1 Introduction

Geospatial data—data with location information—are widely used to inform environmental management and policy decisions, particularly through modeling and mapping exercises. For example, remote sensing data can be paired with ground‐based observations to create terrestrial species distribution maps (Elith and Leathwick 2009a, 2009b). Similarly, global sea surface temperature data and other environmental variables are used in fisheries and marine ecosystem models (Tittensor et al. 2021; Vaihola and Kininmonth 2023), and bathymetry data are used to map geomorphological features on the seafloor (Harris et al. 2014; Yesson et al. 2021). In addition, stakeholder mapping exercises can capture local knowledge of habitat status and ocean uses (Flower et al. 2020; Yates and Schoeman 2013), but require contextual geospatial data—such as bathymetry, seamount locations, and physical and legal boundaries—to act as references.

Before geospatial data can be used in analyses or models, these data must be acquired, processed and standardized (McCarthy et al. 2021). Finding data can be challenging because geospatial data are often scattered across many websites and databases. Acquiring data for a particular area of interest often requires technical skills, such as a knowledge of query languages (e.g., SQL) and database structures. Finally, geospatial data often need to be collated and harmonized to conform to the same spatial units (e.g., spatial grid, point localities of interest, or irregular spatial boundaries) for subsequent analysis, requiring additional geospatial data processing expertise. Although a diverse ecosystem of software has been developed to help with processing geospatial data (e.g., ArcGIS, QGIS, GDAL), the R statistical computing environment (R Core Team 2025) is widely used in the natural and environmental sciences (Lai et al. 2019) and is being rapidly expanded to increase its geospatial processing and visualization capabilities (Lovelace et al. 2025).

Over 20,000 packages are available on the Comprehensive R Archive Network (CRAN 2026) to extend the functionality of the R statistical computing environment. Many of these are designed specifically for geospatial data manipulation (Bivand et al. 2025), such as the *sf* (Pebesma 2018) and *terra* (Hijmans 2025) packages that are widely used for handling vector and raster data. In addition, there are packages that allow for acquisition of specific types of geospatial data, such as *geoData*, principally for terrestrial data (Hijmans et al. 2024), *mregions2* for maritime boundaries (Fernandez‐Bejarano and Pohl 2023), and *biooracler* for marine biophysical data (Assis et al. 2024; Fernandez 2024). However, there are currently no R packages, to our knowledge, that provide access to a broad range of marine geospatial datasets and provide functions for processing and standardizing these data.

Here, we fill this critical gap, introducing the *oceandatr* R package for simplifying the process of acquiring, processing, standardizing, and analyzing marine data. The package provides functions for acquiring many widely used marine datasets, including bathymetry, seamounts, and biophysical data. Additionally, the package provides users with the flexibility to grid and standardize their own data, and classification methods to facilitate visualization and further analyses. Although *oceandatr* is marine focused, its functions to retrieve boundaries, create a custom spatial grid, and aggregate data at the grid level can be applied to terrestrial data. We discuss how the package could provide novel support for various research and management applications, and we offer guidance on how it can be used in conjunction with other R packages. Lastly, we provide case studies where *oceandatr* is used to acquire, grid, and classify data for subsequent use in spatial planning and in a spatial fisheries model.

## 2 Package Specification

The *oceandatr* R package (hereafter, the package) extends the functionality of the R statistical computing environment (R Core Team 2025). The package is designed to be compatible with the existing ecosystem of R packages for processing spatial data and supports the two primary types of geospatial data: raster data in the *SpatRaster* format of the *terra* R package (Hijmans 2025) and vector data in *sf* (Pebesma 2018) format. Details of how to install the package can be found on GitHub where the package is hosted (https://github.com/emlab‐ucsb/oceandatr). The website also contains vignettes demonstrating uses of the package and detailed help pages for each function.

### 2.1 Workflow

**FIGURE 1:**
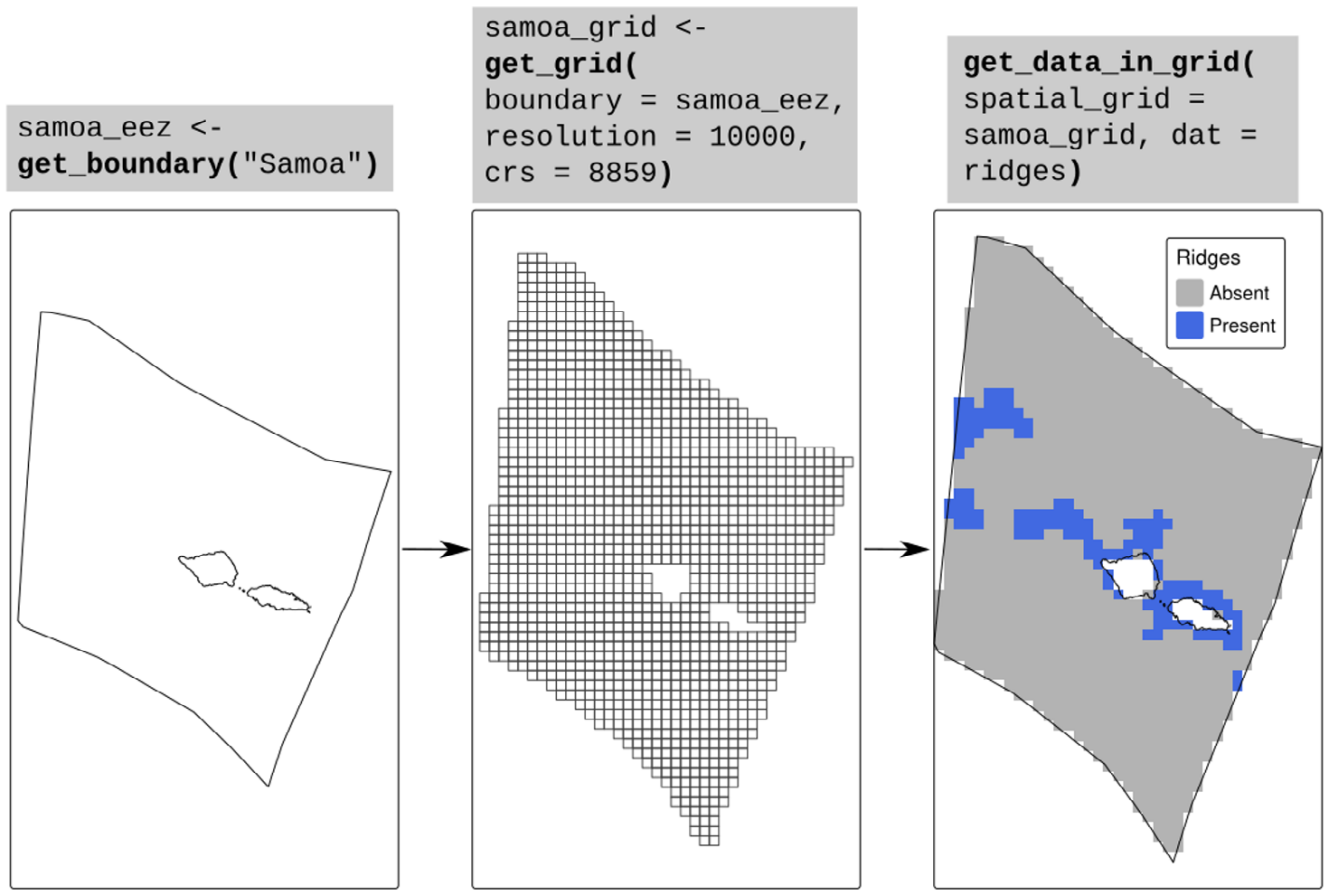
Example usage of the *oceandatr R* package for the Pacific island of Samoa: The EEZ boundary is acquired using the *get_boundary()* function, then a raster grid at a resolution of 10,000 m (10 km × 10 km) covering the EEZ is created using the *get_grid()* function, and data on the distribution of oceanic ridges is placed into that grid using the *get_data_in_grid()* function.

The package has three general functions that: (1) obtain a boundary for an area of interest, (2) create a grid and (3) transfer data into the grid (Figure 1). In addition to these three functions, it has several functions designed to obtain specific datasets (see Section 2.2). The general workflow involves using the function *get_boundary()* to obtain a boundary for the area of interest, such as a country, ocean, or a country's exclusive economic zone (EEZ). To achieve this, the *get_boundary()* function uses the *rnaturalearth* R package (Massicotte and South 2023) to retrieve land boundaries, and the *mrp_get()* function from the *mregions2* R package (Fernandez‐Bejarano and Pohl 2023) to obtain marine boundaries. The advantage of using the *oceandatr* function for retrieving boundary data is that it provides a single function for retrieving both terrestrial and marine boundaries (a capability not currently available in other packages), and the *get_boundary()* function is simpler to use than the *mregions2* functions, which require the user to write a Contextual Query Language filter to select the country or area they want. The boundary can then be used with the *get_grid()* function to create a grid for the area with a specified resolution, coordinate reference system and format. Grids can be created in vector or raster format and can be composed of squares (for raster or vector formats) or hexagons (for vector format). Finally, the *get_data_in_grid()* function is used to transfer vector or raster data into the specified grid. The grid and data supplied to *get_data_in_grid()* can be in different coordinate reference systems, can cross the antimeridian, and can be any combination of raster or vector format. This flexibility is a key strength of the package since data from different sources are often in different formats and poor decisions concerning the spatial transformation and gridding process can result in distorted and inaccurate data. By using the package, users can ensure they have a reproducible and precise approach to processing and standardizing geospatial data.

### 2.2 Data Access

The package provides access to a wide range of geospatial data for the marine environment, including bathymetry and geomorphology; ecoregions and ecology; and human use data (Table 1). To minimize file size downloads, data are downloaded—where possible—in spatial subsets from public servers based on the area of interest. As some data are only available for download as a single, global dataset (i.e., the global seamounts, knolls, coral habitat and geomorphology data), these data are housed in a separate data package, *oceandatrsets*, purpose built to store such data (Flower 2026). This separate data package is automatically installed when installing the *oceandatr* package, and keeping the large datasets separate from the package functions avoids having to re‐download them each time *oceandatr* is updated.

**TABLE 1:** Data accessible through the *oceandatr* package, the function used to access them, and a brief description of the data.

Data	Description	Function	Source	Relevance to spatial modeling and planning
Bathymetry	Global ocean bathymetry data	*get_bathymetry()*	GEBCO 2026 global terrain model (GEBCO Bathymetric Compilation Group 2026) or user provided	Species distributions are strongly depth dependent (Brown and Thatje 2014; Ready et al. 2010)
Depth zones	Ocean depths classified into 5 depth zones: Continental shelf: 0–200 m depth Upper bathyal: 200–800 m depth Lower bathyal: 800–3500 m depth Abyssal: 3500–6500 m depth Hadal: > 6500 m depth	*get_bathymetry(classify_bathymetry = TRUE)*	GEBCO 2026 global terrain model (GEBCO Bathymetric Compilation Group 2026) or user provided. Classification done by package	Depth zones contain different pelagic and benthic habitats (Costello 2009; Vinogradov 1997)
Geomorphology	Global geomorphological features: abyssal hills, abyssal plains, basins, bridges, canyons, escarpments, fans, glacial troughs, guyots, plateaus, ridges, rift valleys, rises, shelf valleys, sills, spreading ridges, terraces, trenches, and troughs	*get_geomorphology()*	www.bluehabitats.org (Harris et al. 2014)	Feature types represent distinct seafloor habitats that are proxies for biodiversity (Ceccarelli et al. 2021)
Seamounts	Global distribution of seamount peaks	*get_seamounts(raw = TRUE)*	Yesson et al. (2021)	Seamounts are known to be biodiversity hotspots (Morato et al. 2010; Rowden et al. 2010)
Seamount peaks buffered	Seamount point locations buffered to a defined distance, i.e., a circle is drawn around each seamount peak	*get_seamounts(buffer = …)*	Yesson et al. (2021)	Morato et al. (2010) found higher biodiversity within 30–40 km of seamounts
Knolls	Global distribution of knolls (base areas). Knolls are small seamounts, < 1000 m but > 200 m above the seafloor (full definition in Morato et al. (2008))	*get_knolls()*	Yesson et al. (2011)	Knolls, as small seamount‐like features, are also likely to host elevated biodiversity relative to surrounding areas
Coral habitat	Predicted ranges of: Antipatharia (black coral), octocorals, and Scleractinia (cold‐water corals) from 3 global distribution datasets	*get_coral_habitat()*	Davies and Guinotte (2011); Yesson et al. (2012, 2017)	Species are vulnerable to damage and are habitat for other species including commercially important fish (Davies and Guinotte 2011; Yesson et al. 2012)
Environmental zones	Environmental zones generated using sea surface data from Bio‐Oracle: chlorophyll concentration (mean), dissolved oxygen concentration (mean), nitrate concentration (mean), pH (mean), phosphate concentration (mean), total phytoplankton (mean), salinity (mean), sea surface temperature (mean, min, and max), and silicate concentration (mean)	*get_enviro_zones()*	Bio‐Oracle data: www.bio‐oracle.org (Assis et al. 2018, 2024). Accessed via the *biooracler* package (Fernandez 2024)	Zones are proxies for different pelagic species assemblages (Magris et al. 2021)
Ecoregions	Global marine ecoregion classifications: Marine ecoregions of the world (Spalding et al. 2007) Longhurst biogeographical provinces (Longhurst 2007) Large marine ecosystems (LMEs) (Sherman and Alexander 1986) Mesopelagic ecoregions (Sutton et al. 2017)	*get_ecoregion()*	Data accessed via the *mregions2* R package (Fernandez‐Bejarano and Pohl 2023)	Ecoregions are commonly used in large‐scale spatial planning to ensure representation of biodiversity (Brito‐Morales et al. 2022; Ceccarelli et al. 2021)
Distance from shore, port or anchorage	Distance from shore, port or anchorage for each grid cell	*get_dist()*	Calculated using shore from Natural Earth (Natural Earth 2024), ports from World Port Index (National Geospatial‐Intelligence Agency 2024) and anchorages from Global Fishing Watch (Global Fishing Watch 2022)	Distance from shore can be used as a proxy for fishing effort (Caddy and Carocci 1999) for small‐scale, nearshore fishing
Fishing effort	Mean total annual fishing effort (hours)	*get_gfw()*	Global Fishing Watch (Kroodsma et al. 2018)	Used as an opportunity cost in spatial planning to avoid excessive fisheries impact (Chollett et al. 2022)

Most of the data *oceandatr* provides access to have global coverage and can be used at a range of spatial scales, from global to country level, for modeling and spatial planning. However, much of the data that the package provides direct access to may not be ideal for analyses at finer spatial scales, such as spatial planning in coastal environments. In these cases, higher‐resolution local data, where available, might be more appropriate. Any spatial data, regardless of whether it was accessed via the package, provided by local experts, or found online, can be prepared using the *get_data_in_grid()* function.

All functions for obtaining data require the user to provide either a spatial grid, in raster or vector format, or a polygon for the area of interest, and can return either gridded data or data within the polygon. The advantage of using the package to obtain data within a user‐provided polygon is that it handles the transformation between coordinate reference systems and can also return data for polygons that cross the antimeridian, a process that can be confusing and potentially lead to problems with the data if not done correctly. If the requested gridded data has multiple data classes (e.g., depth zones in the ocean), a multi‐layer raster will be returned with each class as a separate layer. Conversely, if the grid is in vector format, each class will be a column in the associated data frame. Some functions have further arguments to specify the classification of data, their source, and whether the area of interest crosses the antimeridian. In the following sections, we provide greater detail on the data that can be accessed through the package.

#### 2.2.1 Bathymetric and Geomorphological Data

Bathymetry and geomorphology are widely used in modeling and spatial planning in the marine environment (Brito‐Morales et al. 2022; Harris et al. 2014; McQuaid et al. 2023). Bathymetry data can be retrieved using the *get_bathymetry()* function, and automated routines are available for classifying these data into five commonly used ocean depth zones: continental shelf (0–200 m depth), upper bathyal (200–800 m), lower bathyal (800–3500 m), abyssal (3500–6500 m), and hadal (> 6500 m). By default, these data are retrieved from the GEBCO 2026 global terrain model (GEBCO Bathymetric Compilation Group 2026) hosted at the Natural Environment Research Council's Centre for Environmental Data Analysis (CEDA), allowing access to the latest bathymetry data, a functionality not currently offered by other R packages. To allow downloading of larger‐than‐memory files, the data are downloaded in chunks that are saved sequentially to a single file on the user's hard drive, which is an important capability given the potentially large size of the downloads. Alternatively, *get_bathymetry()* provides the option for the user to specify a local raster file as the source of the bathymetry data, which is useful in cases where local datasets with higher accuracy and resolution are available or the CEDA server is unavailable.

The package has multiple functions to retrieve geomorphological data (Table 1). For instance, the *get_geomorphology()* function returns data from the global seafloor geomorphic features map (Harris et al. 2014). We also include specific functions to access data for seamounts and knolls (smaller seamounts) since these features are known to attract and aggregate biodiversity (Morato et al. 2010; Rowden et al. 2010) and are commonly a key feature for spatial plans (Brito‐Morales et al. 2022; Government of Bermuda and Bermuda Ocean Prosperity Programme 2024). The *get_seamounts()* function provides access to a global seamount dataset (Yesson et al. 2021), while *get_knolls()* can retrieve data on the area of knolls at their base (Yesson et al. 2011). The seamount dataset contains only the peaks of the seamounts (a single point). However, for spatial planning or modeling at local scales, it is useful to define an area around the seamount peak where biodiversity is expected to be higher than the surrounding ocean (Morato et al. 2010). This functionality is available via a “buffer” parameter, whereby the area within a specified radius of the seamount peak is included in the returned polygon or raster. Although the buffer radius can be defined by the user, a 30–40 km buffer radius is recommended as a default value (Morato et al. 2010).

#### 2.2.2 Ecoregions and Ecological Data

Ecoregions are commonly used in spatial planning and modeling to delineate areas of differing environmental conditions (Brito‐Morales et al. 2022; Sala et al. 2021), under the assumption that different conditions support different biological communities. We provide access to four commonly used global ecoregional classification schemes via the *get_ecoregion()* function: marine ecoregions of the world (Spalding et al. 2007); the Longhurst biogeographical provinces (Longhurst 2007); Large Marine Ecosystems (Sherman and Alexander 1986); and mesopelagic ecoregions of the world (Sutton et al. 2017). These data are from the Marine Regions database (https://www.marineregions.org).

Although global ecoregion data are useful at larger scales, regional classifications are likely to be more valuable for planning and modeling at a national scale. The package allows the user to create their own ecoregionalisation, which can be used to delineate environmental zones (i.e., zones based on physical and chemical data). Building on an approach used in Magris et al. (2021), the zones are created using global ocean environmental data from Bio‐Oracle for 11 variables, including sea surface temperature (minimum, maximum and mean), pH and chlorophyll concentration (Assis et al. 2024; Tyberghein et al. 2012). These data are clustered using a kmeans clustering algorithm and the optimal number of clusters is estimated using the *NbClust* R package (Charrad et al. 2014), but can also be specified by the user. Each cluster represents a unique environmental ‘zone’. Both the raw environmental data and the clustered environmental zones can be retrieved using the *get_enviro_zones()* function.

The package can also access output from a limited number of species distribution models (SDMs) or habitat suitability models that predict the spatial distribution of species of interest. These models are commonly used in spatial planning as biodiversity layers to inform which areas to protect. While global SDMs for marine species exist (O'Hara et al. 2017), these require manual download for each species and cannot be included in the package due to usage restrictions and file size constraints. However, global cold water coral habitat suitability maps can be accessed for the species groups Antipatharia (Yesson et al. 2017), octocorals (Yesson et al. 2012) and Sclerarctinia (Davies and Guinotte 2011) using the *get_coral_habitat()* function. For inclusion in spatial planning, it is common to convert habitat suitability values to presence‐absence format. The original Scleractinia data are made available in presence‐absence format, while the habitat suitability values for Antipatharia are converted to presence‐absence by the package using the threshold value proposed by the dataset authors (Yesson et al. 2017), and octocorals are considered present in cells where at least 2 octocoral suborders are present (range of values: 0–7), though these thresholds can be changed by the user.

#### 2.2.3 Human Use

Data on human use of the ocean is used extensively in spatial planning and fisheries and ecosystem models (Frawley et al. 2022; Lotze et al. 2019; Sala et al. 2021). In spatial planning, maps of fishing intensity often serve as a proxy for the opportunity cost of marine protection (Chollett et al. 2022). A widely used source of these data is Global Fishing Watch (GFW), which provides global data on apparent fishing effort (hours of fishing) at hourly, 0.01° resolution based on automatic identification system (AIS) data, a satellite tracking system for large vessels (Kroodsma et al. 2018). The *gfwr* R package allows access to many GFW data products via an API key (Clavelle et al. 2024), but fishing effort data can only be obtained for a single year per query. The *get_gfw()* function in *oceandatr* obtains GFW fishing effort data for multiple years and allows users to sum or average the data across selected years, but still requires an API key, which can be created for free on the GFW website (https://globalfishingwatch.org).

Only larger, industrial‐scale vessels are required to use AIS, and thus these data may not reflect fishing effort in areas where fishing is mainly by small, nearshore fishing vessels. In these cases, users could import other sources of fishing effort data if available. If no such data are available, distance to port or shore may be a reasonable proxy for fishing effort (Caddy and Carocci 1999). The *get_dist()* function can be used to get gridded distance from ports, anchorages, or shore. Port data are downloaded directly from the World Port Index, which is updated monthly (National Geospatial‐Intelligence Agency 2024), and the distance to shore is calculated from the Natural Earth country boundaries layer (Natural Earth 2024). Anchorages are from GFW, which includes > 167,000 geospatial points (Global Fishing Watch 2022). As calculating distance from ports and anchorages to each grid cell is computationally expensive, *oceandatr* provides two derived datasets. One groups anchorages with the same name into a single point, which is then assigned the mean longitude and latitude coordinates from the aggregated points. The second takes this aggregated dataset and removes anchorages that fall within land boundaries of countries, as defined by Natural Earth data (Natural Earth 2024), such as ports on rivers, assuming that users of the package will be principally interested in distance to coastal ports.

## 3 Package Usage

The package can acquire and process bathymetric, biophysical, and geomorphology data that are suitable for use in multiple modeling, mapping, and spatial planning contexts (Table 2). For example, data acquired using the package could be used to model fishing fleet behavior and effort distribution (see Section 3.2) or model species distributions in conjunction with observation data. Furthermore, all data acquired via the package are suitable for use in marine spatial planning using prioritization tools, such as the *prioritizr* R package (see Section 3.1). Data can also be used for site selection in industry applications, such as aquaculture and renewable energy, and for mapping for stakeholder engagement processes. More broadly, the *sf* vector data and *SpatRaster* raster data formats used as inputs and outputs by the package functions are widely used and can therefore be easily integrated into other spatial data processing and analysis workflows.

**TABLE 2:** Example usage of the package, and how it could complement existing R packages in data analysis.

Use case	How *oceandatr* can be used	Other R packages that could aid analysis
Species distribution models	Retrieve gridded data to use as predictors of species presence or abundance	*robis* (Provoost et al. 2022) and *rgbif* (Chamberlain et al. 2025) for accessing species occurrence data. *ENMeval* (Kass et al. 2021) for model fitting and tuning, *inlabru* (Bachl et al. 2019) for Bayesian spatial modeling
Fishing vessel behavior models	Retrieve gridded physical and environmental data to be used as explanatory variables and fisheries data as a response variable	*sdmpredictors* (Bosch and Fernandez 2023) enables download of additional environmental data. *mgcv* (Wood 2017) for modeling
Spatial planning: conservation planning	Retrieve gridded and classified data that can be used as inputs in a spatial prioritization	*prioritizr* (Hanson et al. 2025) for running a spatial prioritization
Spatial planning: site selection for aquaculture, marine renewable energy, and other industries	Retrieve data used in geospatial analysis such as multi‐criteria evaluation models	*ipdw* (Stachelek 2023) for interpolating point data into raster layers
Stakeholder consultation and participatory mapping	Retrieve data for use in maps that can aid stakeholder discussions about MSP	*tmap* (Tennekes 2018) and *ggplot* (Wickham 2016) for creating maps. Shiny (Chang et al. 2025) for interactive web applications

### 3.1 Case Study: Spatial Conservation Planning

The following case study illustrates the use of the package to acquire and process geospatial data that is then used directly to identify priority areas for protection. This type of conservation planning exercise requires several inputs: a planning region (area of interest), features of interest (e.g., species or habitats) that can be conserved by selecting planning units (spatial units within the planning region), cost data for each planning unit, and a target amount of each feature to conserve within selected planning units (Ardron et al. 2010). The cost data can be the economic cost of protecting a planning unit (e.g., money required to buy spatial area delineated by the planning unit), but in many contexts, especially ocean planning, opportunity cost is used, i.e., the value of each planning unit to fisheries assuming fishing would be prohibited in protected locations. After assembling these data, they can be input into a conservation planning decision support tool, such as the *prioritizr* R package (Hanson et al. 2025) and, in turn, used to identify priority areas for protection or implementation of conservation management actions.

**FIGURE 2:**
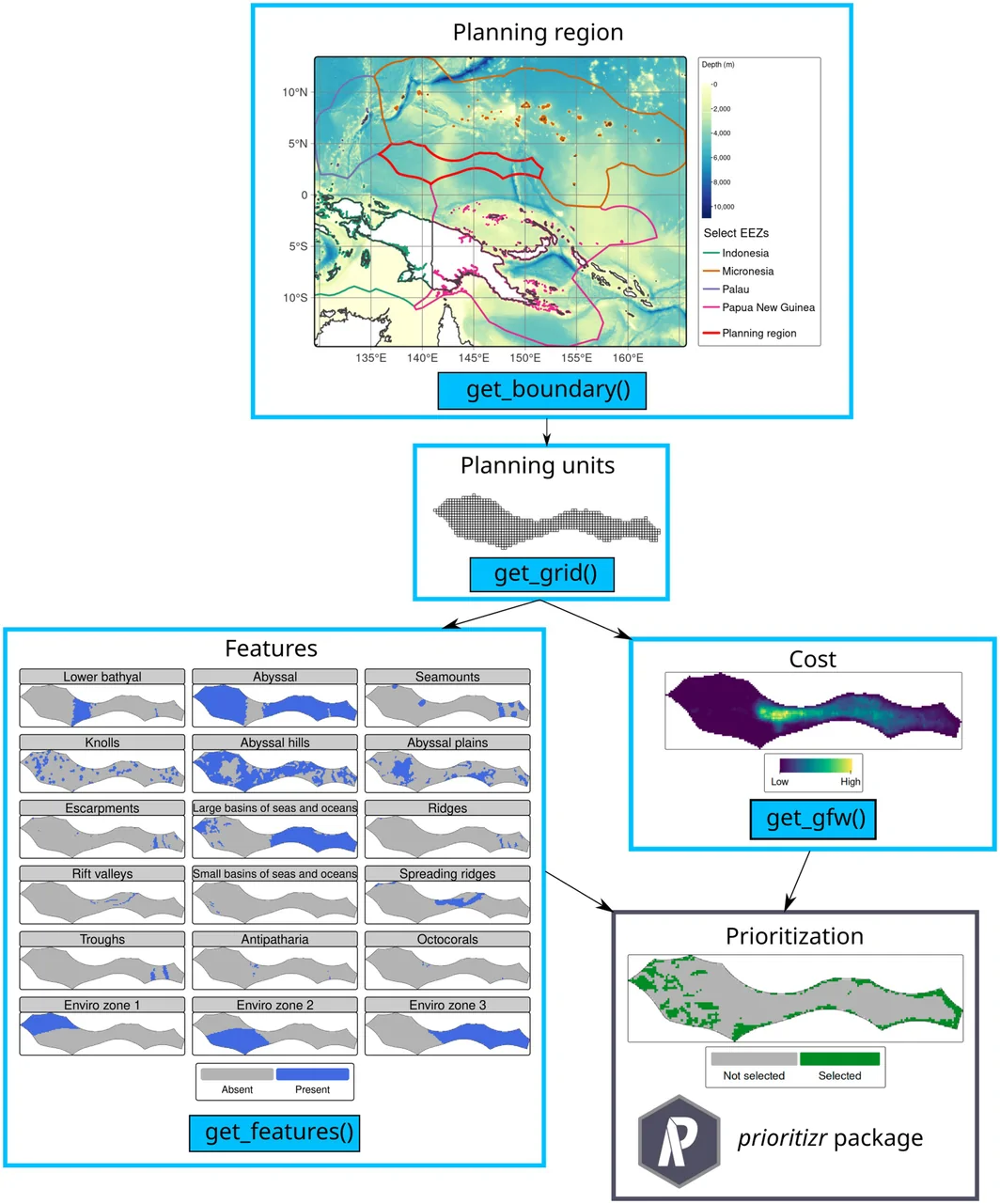
Workflow for using the oceandatr package to acquire and process data (blue boxes) to generate a prioritization with the *prioritizr* package (gray box). The workflow has five stages: (i) defining the planning region, (ii) generating planning units, (iii) generating feature data, (iv) generating cost data, and (v) generating a prioritization. Each box denotes a stage of the workflow, and maps are the output from each stage. Blue text boxes show the *oceandatr* functions associated with each stage. Depth data shown in the map of the planning region is from the GEBCO 2026 global terrain model (GEBCO Bathymetric Compilation Group 2026) accessed using the *get_bathymetry()* function in *oceandatr*.

To demonstrate the utility of *oceandatr* in a conservation planning workflow, we use a high seas area of the Pacific Ocean as a case study (Figure 2). We created the polygon for the planning region using the *get_boundary()* function to obtain all high seas areas and then cropped the polygon to the part of the Pacific of interest (~0°–6°N and 135°–155°E, Figure 2). Next, we used the *get_grid()* function to create a spatial grid (comprising 10 km × 10 km grid cells) to serve as planning units for the case study. The spatial grid was then passed to the *get_features()* function which calls each of the separate functions to obtain data on bathymetry, geomorphology, buffered seamount peaks, knolls, coral habitat and environmental zones, and returns a multi‐layer raster or multi‐attribute vector. We used apparent fishing effort as the cost, using the *get_gfw()* function to obtain the total annual fishing effort for 2022. Another option would be to use distance from shore or port through the *get_dist()* function, with the assumption that areas closer to the coast or port have greater fishing effort since they are more easily accessible. On an Intel Core i7‐8700T CPU@2.40GHz Desktop PC with 6 cores and 16GB of memory, the data retrieval and processing workflow using *oceandatr* took 2 min and 16 s, resulting in a total of 19 raster layers with 8142 cells (planning units). The elapsed time will vary depending on internet connection speed, computer processing power, and the resolution of the data created.

The data obtained using *oceandatr* can be used directly in *prioritizr* and other spatial prioritization tools such as Marxan (Ball et al. 2009) and Zonation (Moilanen et al. 2009). For this example, we used targets of 20% of the area for each feature to conserve, although any target between 0 and 100% can be used, and *prioritizr* yields a solution (the priority areas for protection; Figure 2). The same workflow can be used to quickly and simply create a spatial prioritization for any ocean area. Data that are not part of the *oceandatr* package can be prepared for prioritization using the *get_data_in_grid()* function. This function intersects planning units with the provided data, automatically projecting data into the desired coordinate reference system, and could be used for data preparation for terrestrial and freshwater spatial prioritization contexts.

### 3.2 Case Study: Modeling Fishing Effort

This case study demonstrates the use of *oceandatr* to obtain and standardize human use and environmental data for modeling fishing effort. Numerous factors drive the behavior of fishing fleets and where they allocate fishing effort, including weather conditions (Lehodey et al. 2006), distance from port (Caddy and Carocci 1999), and the distribution and abundance of the target species. Understanding the relationship between physical and environmental factors and fishing effort is important for resource management (Vaihola and Kininmonth 2023). There are many models and modeling frameworks available, but in this example we use a Generalized Additive Model (GAM), which accommodates non‐linear environmental relationships and inherent spatial autocorrelation and is widely used for spatial modeling in fisheries contexts (Mugo et al. 2010; Stock et al. 2020; Vaihola and Kininmonth 2023).

**FIGURE 3:**
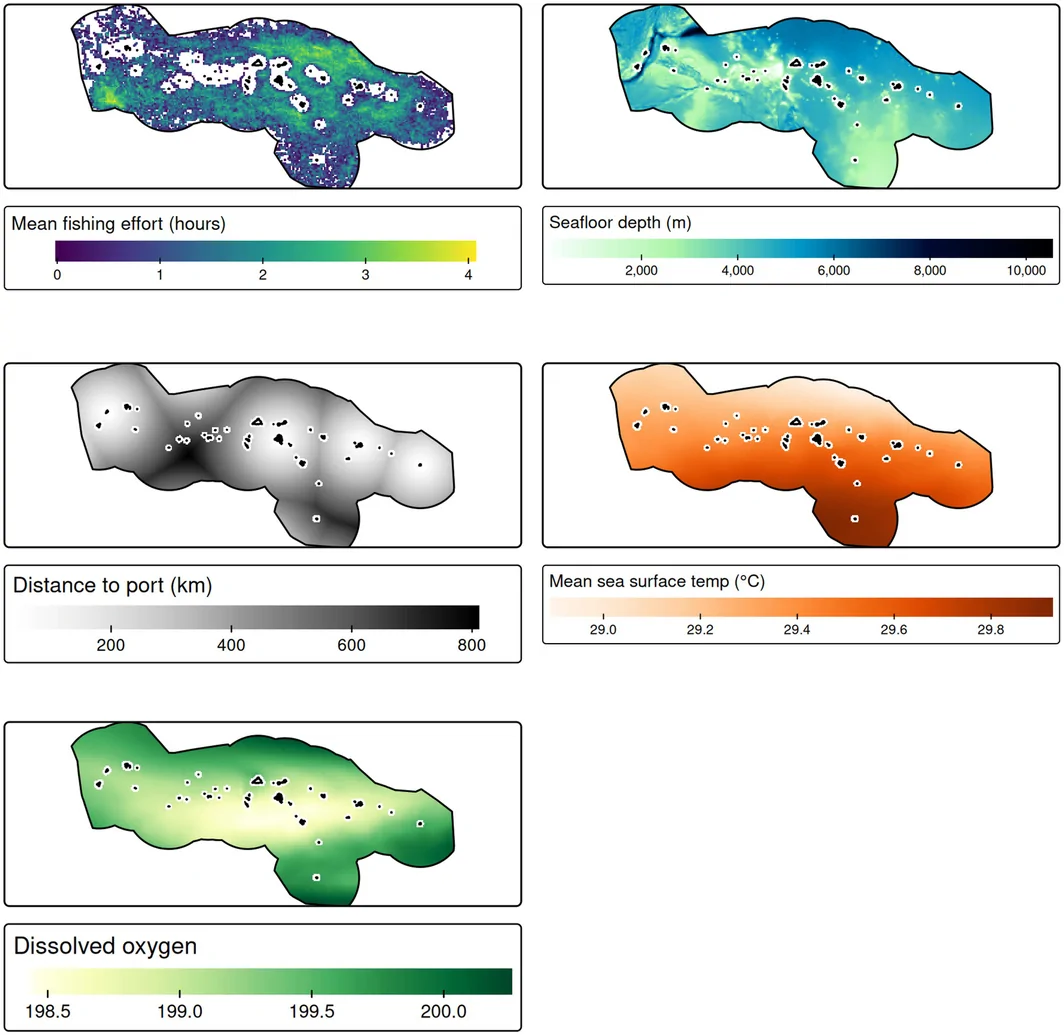
Spatial data for the Federated States of Micronesia's EEZ used in the Generalized Additive Model. Mean fishing effort is the response variable, while seafloor depth, distance to port, mean temperature, and dissolved oxygen are the predictors. All data were obtained and gridded using *oceandatr* functions.

The workflow was similar to that in the first case study (*3.1* *Case study: Spatial Conservation Planning*). Spatial boundaries for the area of interest were obtained using the *get_boundary()* function, a spatial grid was created using *get_grid()*, and data were obtained using relevant functions (e.g., *get_bathymetry* and *get_enviro_zones()*). We chose the Federated States of Micronesia (FSM) as the area of interest, because it has extensive Global Fishing Watch fishing effort data available for its exclusive economic zone (EEZ). The EEZ boundary was obtained using *get_boundary(“Micronesia”)*, and the *get_grid()* function was used to create a spatial grid (comprising 10 km x 10 km grid cells) for all the model data. We downloaded and standardized all data needed for the modeling process using: *get_gfw()* to obtain mean annual fishing effort for 2024–2025 from Global Fishing Watch, *get_bathymetry()* and *get_dist()* to obtain seafloor depth and distance from port respectively, and *get_enviro_zones()* with the argument *enviro_zones = FALSE* to obtain sea surface environmental data, including mean temperature, pH and chlorophyll‐a concentration. The environmental data were subset to only the mean sea surface temperature (SST) and dissolved oxygen concentration data because these influence tuna distributions (Vaihola and Kininmonth 2023) which is the principal target species for fishing fleets in the FSM (Bell et al. 2021). All data were then combined into a single, multi‐layer raster (Figure 3), and converted to a dataframe ready for the GAM.

**FIGURE 4:**
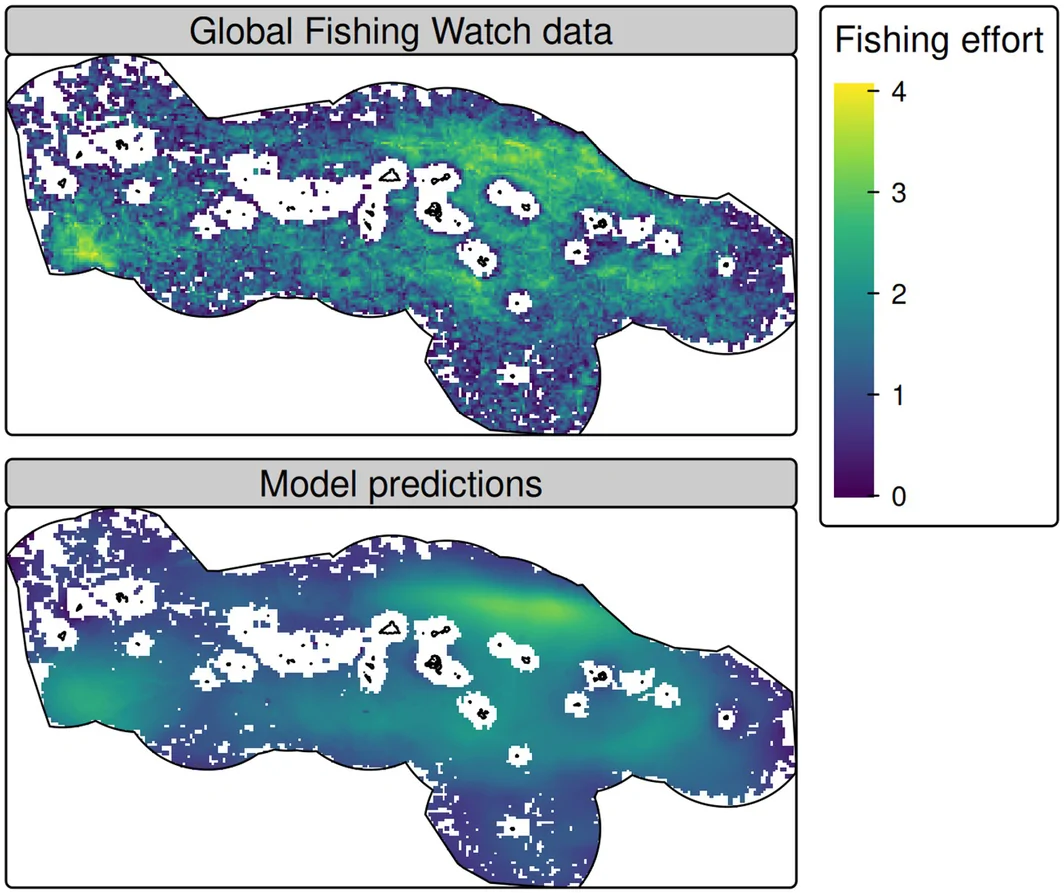
Comparison of GAM predictions of fishing effort to fishing effort data from Global Fishing Watch that was the model response variable.

We created a GAM using the *gam()* function in the package *mgcv* (Wood 2017). Predictor variables (seafloor depth, distance to port, SST and dissolved oxygen) were modeled using penalized thin‐plate regression splines, while a geographic Gaussian process smoother was integrated to account for spatial dependency across the spatial grid. The model was fitted via Restricted Maximum Likelihood (REML) estimation using the log+1 transformed response (fishing effort) variable to address data skewness and stabilize residual variance, and we used a normal error structure. We fitted only a single GAM with the four predictor variables because this case study was intended to demonstrate the functionality of *oceandatr*, although we acknowledge that a more rigorous modeling process would include variable selection procedures (e.g., via the *MuMIn* package, Bartoń 2026). The resulting model successfully captured the spatial distribution of fishing effort (adjusted *R* ^2^ = 0.49) and all predictor variables had highly significant, non‐linear effects (*p* < 0.001). We used the *predict.gam()* function from the *mgcv* package to predict fishing effort in FSM to visually compare the Global Fishing Watch fishing effort data with fishing effort predicted by the GAM (Figure 4).

The case study illustrates the ease with which spatial modeling can be done using *oceandatr* to obtain standardized data ready for the modeling process. The code to repeat the workflow outlined here is available on the *oceandatr* website as a vignette (https://emlab‐ucsb.github.io/oceandatr/articles/fishing_prediction.html).

## 4 Conclusion

Acquiring, processing and standardizing geospatial data requires technical skills and considerable time. The o*ceandatr* R package helps lower the barrier to entry for ocean‐focused geospatial work by providing a single package to acquire and process many widely used datasets. Processed data is stored in the *SpatRast* format of the *terra* R package (Hijmans 2025); and vector data in *sf* (Pebesma 2018) format, facilitating mapping or further spatial analysis. We recognize that in many cases, data that are not directly accessible via *oceandatr* might be required. The *get_data_in_grid()* function is designed for these cases since it can process arbitrary spatial data. Along with the *get_boundary()* and *get_grid()* functions, *get_data_in_grid()* can be used in any spatial context, not just for the marine environment.

While some of the data that *oceandatr* provides access to are available through other R packages or direct download from websites, *oceandatr* provides a single access point for many of the most commonly used marine datasets. In addition, the functions provide a simpler interface to some of the datasets than existing packages. The *oceandatr* package is also the only R package, to our knowledge, providing direct download of spatial subsets of the latest global terrain model (GEBCO Bathymetric Compilation Group 2026), which is widely used in marine data analysis.

We have focused on providing access to data commonly used in marine spatial analysis, such as bathymetry, geomorphology, and fishing data. While there are several other datasets that are widely used, *oceandatr* is restricted to data that are open access and can be easily downloaded from the internet or are small enough to be included within the associated *oceandatrsets* data package (Flower 2026). These criteria currently exclude the IUCN (IUCN 2024) and Aquamaps (Kaschner et al. 2019) species distribution maps, which are widely used in spatial planning, because both ask users to contact the data authors before use. However, once obtained, these data can be processed for modeling or planning work using the *get_data_in_grid()* function.

The *oceandatr* package is currently being used by the authors in marine spatial planning contexts to rapidly and reproducibly obtain standardized data for spatial prioritization, as exemplified in the first case study. The package will therefore be continuously improved to increase the number of datasets directly accessible, improve data processing efficiency, and add further documentation.

## Author Contributions

**Jason Flower:** conceptualization (lead), data curation (lead), funding acquisition (lead), methodology (lead), software (lead), supervision (lead), writing – original draft (lead), writing – review and editing (lead). **Echelle S. Burns:** data curation (supporting), methodology (supporting), software (supporting), writing – original draft (supporting), writing – review and editing (supporting). **Daniel C. Dunn:** funding acquisition (supporting), methodology (supporting), writing – original draft (supporting), writing – review and editing (supporting). **Andy Estep:** conceptualization (supporting), funding acquisition (supporting), writing – original draft (supporting). **Jason D. Everett:** methodology (supporting), software (supporting), writing – original draft (supporting), writing – review and editing (supporting). **Jeffrey O. Hanson:** methodology (supporting), writing – original draft (supporting), writing – review and editing (supporting). **Sarah E. Lester:** funding acquisition (supporting), methodology (supporting), supervision (supporting), writing – original draft (supporting), writing – review and editing (supporting). **Anthony J. Richardson:** funding acquisition (supporting), writing – original draft (supporting), writing – review and editing (supporting).

## Funding

This work was funded by the Waitt Foundation.

## Conflicts of Interest

The authors declare no conflicts of interest.

## Data Availability

The *oceandatr* package is freely available from Github: https://github.com/emlab‐ucsb/oceandatr. The associated website (https://emlab‐ucsb.github.io/oceandatr/) has several tutorials, including the two case studies in this paper. A released and archived version of the package, used in the preparation of this paper, is available on Zenodo: https://doi.org/10.5281/zenodo.21149583. The associated data package, *oceandatrsets*, is also available on Github: https://github.com/emlab‐ucsb/oceandatrsets.
